# Sexual self-efficacy and sexual quality of life before and after laparoscopic surgery in women with endometriosis lesions: A cross-sectional study

**DOI:** 10.18502/ijrm.v20i6.11442

**Published:** 2022-07-06

**Authors:** Soraya Malekmaleki, Shadab Shahali, Ashraf Moini

**Affiliations:** ^1^Department of Reproductive Health and Midwifery, Faculty of Medical Sciences, Tarbiat Modares University, Tehran, Iran.; ^2^Department of Endocrinology and Female Infertility, Reproductive Biomedicine Research Center, Royan Institute for Reproductive Biomedicine, ACECR, Tehran, Iran.; ^3^Department of Obstetrics and Gynaecology, Tehran University of Medical Sciences, Tehran, Iran.; ^4^Department of Obstetrics and Gynaecology, Arash Women's Hospital, Tehran University of Medical Sciences, Tehran, Iran.

**Keywords:** Laparoscopy, Endometriosis, Dyspareunia, Self-efficacy, Quality of life.

## Abstract

**Background:**

Endometriosis is one of the most common gynecological diseases and is associated with an increased risk of dyspareunia.

**Objective:**

This study aimed to evaluate sexual self-efficacy (SSE) and sexual quality of life (SQOL) before and after laparoscopic surgery of endometriosis lesions.

**Materials and Methods:**

This cross-sectional study measured the dependent variable by questionnaires before and after the surgery, and was performed with 36 women with endometriosis (aged 20-40 yr) who were referred to Arash hospital, Tehran, Iran between December 2018 and July 2019 using a convenience sampling method. Data collection tools included SQOL and SSE questionnaires, and a visual analogue scale. Data were collected before, and 3 and 6 months after laparoscopic surgery.

**Results:**

Out of the 36 women included in this study, 91.7% had pelvic endometriosis and 8.3% had abdominal and pelvic endometriosis. 9 participants (25.0%) were in stages I or II of the disease, and 29 (75.0%) had endometriosis with higher involvement. The findings of the study showed a positive effect of laparoscopic surgery on SQOL and SSE in participants (p < 0.001).

**Conclusion:**

Laparoscopic surgery in cases of endometriosis can improve SQOL and SSE in affected women and improve dyspareunia.

## 1. Introduction

Endometriosis is an estrogen-dependent inflammatory disease, in which endometrial cells are found somewhere outside the uterine cavity (1). Although many endometriosis patients are asymptomatic, they may experience menstrual pain, intercourse pain, and chronic pelvic pain (2). Endometriosis is one of the most common gynecological diseases. In 2012, its prevalence was estimated to be 5-20% among women of childbearing age in Tehran (3). Severe pelvic pain is often associated with endometriosis, and this pain can be alleviated by treatments that suppress estrogen production. Endometriosis is commonly seen in the pelvis, ovaries, cul-de-sac, uterine ligament, pelvic peritoneum, and rectovaginal septum. Extrauterine endometriosis occurs when endometriosis lesions are found elsewhere in the body, such as in the cervix, vulva, vagina, intestine, urinary system, abdominal wall, chest, lung, and central nervous system (4). Several studies have reported an association between endometriosis and sexual dysfunction (5-7). The severity of the endometriosis is also directly related to the deep dyspareunia severity (8). Dyspareunia is also associated with other forms of sexual disorders due to fear of pain (9).

Endometriosis is treated using a variety of methods, divided into 2 categories: medical and surgical treatments (10). Medical treatments seek to achieve hypoestrogenic status in the patient and to reduce patient pain but are associated with the possibility of recurrence after discontinuation; moreover, the primary goal in surgical treatment is to eliminate the whole disease and its associated symptoms (11). It is now known that medical treatment alone is insufficient, and surgical intervention is needed in these patients (12).

Some studies have suggested that laparoscopy not only has a positive effect on recovery and dyspareunia, but also can improve the individual's sexual quality of life (SQOL) (13-15). Endometriosis is known to increase the chance of deep dyspareunia, which can have negative consequences for overall female sexual functioning and couple relationships (15). Previous studies that have compared the medical and laparoscopic therapies of this disease have focused on the therapeutic aspect of endometriosis and have neglected the impact of this disease on other aspects of life (16-17). Also, the limited previous studies that have studied the impact of this disease on sexual life have only examined the effects of this disease on specific issues such as sexual satisfaction and SQOL (13, 11, 14-15, 18-19). No studies have examined the effect of this disease on sexual self-efficacy (SSE), which is one of the most important components of having a successful marital relationship; these women may develop different ways to cope with the experience of pain and recurrent loss of desire and orgasm.

Therefore, the present study aimed to evaluate the differences in SSE and SQOL before and after laparoscopic surgery of endometriosis lesions.

## 2. Materials and Methods

### Study setting and participants

This cross-sectional study was conducted at Arash hospital, Tehran, Iran between December 2018 to July 2019. The sample size was calculated based on female SQOL before and 3 months after laparoscopic surgery by comparing the following means formula: 


$$n={\left[\frac{(z_{\alpha /2}+z_{\beta })S}{d}\right]}^{2}$$

where type I error α = 0.05, type II error β = 0.2, Z_ (1-α⁄2) = 1.96, Z_ (1-β) = 0.84, average difference d = 2.1 and S = 2.8, based on the results of a similar study (20), which was a pilot study conducted by the research team.

The necessary sample size was thus determined to be 36 persons, given a predicted drop-out rate of 10%. Participants were women who were referred to Arash hospital, Tehran, Iran for endometriosis laparoscopy. The recruitment was through the non-random convenience method.

### Inclusion and exclusion criteria

The inclusion criteria were: women aged 18-40 yr who were candidates for laparoscopic surgery for their endometriosis lesions, which were diagnosed by a gynecologist with no limitation of duration of endometriosis; ability to write and speak in Persian; first marriage; and a history of sexual activity during the past month. The exclusion criteria were: known infertility problem (infertility has a strong impact on a woman's psychosexual behavior and overall quality of life, so as it was a confounder in this study, we excluded these women); a history of mental illness or chronic disease, based on the patient's file, including diabetes, heart disease, kidney disease, connective tissue disease, pelvic inflammatory disease, or grade 3 or 4 pelvic prolapses; use of combined oral contraceptive pills, gonadotropin-releasing hormone analogs, danazol or related drugs within 6 months before surgery; use of antidepressants or other medications that can affect sexual function (e.g., beta-blockers, antihistamines, antipsychotics, benzodiazepines, antiepileptics); smoking; or alcohol or drug substance use disorder.

### Primary outcome assessment

Before laparoscopic surgery, an SSE questionnaire, the sexual quality of life-female questionnaire, and a demographic information questionnaire (which collected data on age, gender, occupational status, etc.) were completed by all participants. The severity of dyspareunia was also assessed by the visual analogue scale.

The SSE questionnaire used was Vaziri's SSE scale (designed based on the Schwartz SSE Questionnaire), which consisted of 10 questions and was graded in a 4-choice continuum from zero (completely incorrect) to 3 (completely correct). The SSE scores were divided into 3 categories: low (0-10), moderate (10-20), and high (20-30). The validity of the SSE questionnaire in Iran was assessed by Vaziri and Kashani using the content validity method. The reliability of this questionnaire for total scores was found to be 0.851 using Cronbach's alpha method and 0.817 using the split-half method (21).

The sexual quality of life-female questionnaire was designed by Simmonds et al. (22) and it contained 18 items categorized into the 4 themes of sexual psychology, sexual and marital satisfaction, self-worthlessness, and sexual repression. Assessment was based on a 6-point Likert scale (strongly agree to strongly disagree) with scores ranging from 0-5 or 1-6 for every item and a possible score range of 0-90 or 18-108. A higher score indicated better QOL. The validity and reliability of the Persian version of this questionnaire were assessed by Masoumi and colleagues. To determine validity, the content validity index (0.91) and content validity ratio (0.84) were used, and to determine the reliability, the internal consistency coefficient Cronbach's alpha (0.73) and intra-cluster correlation index (0.88) were used (23).

The subjects were followed up 3 and 6 months after their surgery and the questionnaires were completed either at the time of referral to the hospital or by phone. Pathology results were followed up and recorded.

### Ethical considerations

Ethical approval for this study was obtained from the Tarbiat Modares University Ethics Committee, Tehran, Iran (Code: IR.MODARES.REC.1397.152). The objectives of the study were explained to the participants, and all participants provided informed written consent.

### Statistical analysis

Data analysis was performed using the Statistical Package for the Social Sciences (SPSS) statistical program, version 22 (IBM Corp., Armonk, NY, USA). Repeated measure ANOVA was used to compare the data of the quantitative variables before and after the laparoscopic surgery. Pearson and Spearman correlations were used to assess the relationships between the variables. Descriptive data were analyzed using absolute and relative frequency distributions, mean and standard deviation (SD). P < 0.05 was considered as the level of significance.

## 3. Results

All 36 participants were followed up until the end of the study. The mean age of the participants was 33.13 ± 4.69 yr (range of 20-40 yr), 66.67% (n = 24) of the study population were housewives and 55.55% (n = 20) had a tertiary education. Household income was estimated to be moderate in 77.78% of the participants (n = 28). Baseline demographic characteristics of the participants are shown in table I.

All participants were fertile and had active sex with their husbands, 20 of whom were nulliparous (55.55%) and 16 (44.45%) were multiparous. Half of the participants (n = 18, 50.00%) had experienced a c-section, 15 (41.66%) had experienced a vaginal delivery, and 3 (8.34%) had experienced both. 21 (58.34%) of the subjects had used withdrawal as a birth control method and 15 (41.66%) had used condoms. Based on the evaluation of the participants, 8 of the women had a dyspareunia severity score of 6 (22.23%), 10 had a score of 7 (27.78%), 12 had a score of 8 (33.33%), and 6 had a score of 9 (16.66%).

9 (25.00%) of the subjects were in stages I or II of the disease, and 75.00% (n = 27) were in stages III or IV of endometriosis. Participants were also evaluated based on sites of endometriosis, with 91.66% (n = 33) having pelvic endometriosis and 8.34% (n = 3) having abdominal and pelvic endometriosis.

According to the post-surgical follow-up of participants, none of the participants had any surgical complications such as adhesion or bleeding during the follow-up period.

The results of the study obtained by repeated measure ANOVA and the post hoc test on participants' SSE indicated that the mean ± SD of pre-surgical SSE was 7.41 ± 6.45, while it was 13.47 ± 7.47 3 months after surgery (p ≤ 0.001) and 6 months later it reached 17.77 ± 9.49 (p ≤ 0.001). The mean ± SD of SQOL increased from 37.08 ± 10.06 before the surgery to 48.66 ± 11.88 3 months after surgery (p ≤ 0.001) and 6 months later it reached 53.36 ± 15.91 (p ≤ 0.001). Both of these showed a significant increase in mean postoperative value compared to mean preoperative value (Table II). At the 3-month follow-up, 27.7% of participants did not have dyspareunia, 66.6% reported a decrease in dyspareunia ( > 20 mm decrease in the visual analogue scale), and no change was observed in dyspareunia in 5.7% of the participants. At the 6-month follow-up, 75.0% reported no dyspareunia.

There was an inverse correlation between SQOL (r = -0.45, p = 0.01) and SSE (r = -0.35, p = 0.03), and endometriosis involvement. There was no significant correlation between age, educational level, income level, contraception methods, age of menarche, or site of endometriosis with SQOL or SSE according to the Spearman and Pearson correlation tests (Table III).

**Table 1:** Characteristics of research subjects


**Demographic variables**		**Number (%)**
**Level of education**		
	**Primary**	5 (13.89)
	**Secondary**	11 (30.56)
	**Tertiary**	20 (55.55)
**Education level of spouse**		
	**Primary**	3 (8.33)
	**Secondary**	4 (11.11)
	**Tertiary**	29 (80.56)
**Employment status**		
	**Housewife**	24 (66.67)
	**Administrative**	10 (27.77)
	**Specialist**	2 (5.56)
**Employment status of spouse**		
	**Administrative**	21 (58.33)
	**Worker**	13 (36.11)
	**Retired**	2 (5.56)
**Economic status of the family**		
	**Poor**	4 (11.11)
	**Medium**	28 (77.78)
	**Rich**	4 (11.11)
**Contraception**		
	**Condom**	15 (41.66)
	**Withdrawal method**	21 (58.34)

**Table 2:** Scores of sexual quality of life and sexual self-efficacy of subjects before, 3 months and 6 months after endometriosis laparoscopy


**Variable**	**Before surgery **	**3 months from the beginning of the study**	**6 months from the beginning of the study**	**P-value**
**Sexual quality of life**	37.08 ± 10.06	48.66 ± 11.88	53.36 ± 15.91	before vs. 3 months: 0.007* 3 months vs. 6 months < 0.001 before vs. 6 months ≤ 0.001
**Sexual self-efficacy**	7.41 ± 6.45	13.47 ± 7.47	17.77 ± 9.49	before vs. 3 months < 0.001 3 months vs. 6 months < 0.001 before vs. 6 months < 0.001
Data presented as Mean ± SD. Repeated measure ANOVA test and post hoc test results. P < 0.05 was considered significant				

**Table 3:** Correlation between sexual quality of life, sexual self-efficacy and characteristics of research subjects


**Variables**	<Stage of endometriosis*		<Site of endometriosis*		<Age**		<Educational level*		<Income level*		<Contraception method*		<Age of menarche**	
	**r**	**P-value**	**r**	**P-value**	**r**	**P-value**	**r**	**P-value**	**r**	**P-value**	**r**	**P-value**	**r**	**P-value**
**Sexual** **quality** **of life**	-0.45	0.00	-0.30	0.07	0.00	0.99	0.19	0.26	0.11	0.48	0.14	0.40	0.19	0.25
**Sexual** **self-efficacy**	-0.35	0.03	-0.36	0.05	-0.06	0.71	0.31	0.06	0.04	0.79	0.19	0.22	0.18	0.27
*Spearman correlation test, **Pearson correlation test, P < 0.05 was considered significant														

## 4. Discussion 

Patients with endometriosis may develop severe pain associated with the site of their involvement and severity of disease, including dyspareunia, dysmenorrhea, dysgenesis, and painful bowel movements that can affect their SQOL (1, 5-7).

The findings of our study showed a positive effect of laparoscopic surgery on SQOL and SSE in our participants. Numerous studies have reported a relationship between pain during sexual intercourse and anxiety, less frequent or even avoiding intercourse, lower levels of desire and arousal, and orgasmic disorder, which can have negative effects on women's physical and psychological well-being and may disrupt couples' relationships (5-7). Some studies have shown that laparoscopic surgery not only can have a positive effect on the recovery rate and dyspareunia but also can improve SQOL (11, 15, 24). For example, Fritzer and co-authors demonstrated that laparoscopy improved SQOL in the participants of their study and reduced pain (11). A prospective case-control study found that participants' SQOL was improved and pain and discomfort were decreased 6 months after surgery, with a modest reduction at 36 months (15). A comparative study on women with deep endometriosis undergoing laparoscopic surgery vs. women undergoing nerve-sparing surgery, showed that sexual satisfaction scores improved 24 months after surgery in both groups, although these were still lower than normal sexual satisfaction scores (24). Furthermore, Ferrero et al. (18) showed that laparoscopy resulted in a significant decrease in the severity of dyspareunia and a complete loss of pain sensation in 80.6% of their subjects.

According to our results, the mean (SD) of preoperative SSE was 7.41 (6.45), which increased to 17.77 (9.49) 6 months after surgery, and it was observed that laparoscopic surgery had a positive effect on SSE. Another study showed that we can eliminate the underlying sexual problems of women by increasing their SSE, so that the higher the SSE, the better the sexual function; also, the higher the SSE, the better the ability to resolve sexual problems (e.g., dyspareunia) (25).

The results of the present research demonstrated that the mean score of SQOL was improved by 6 months after surgery, which indicated a positive effect of laparoscopy on SQOL. This finding is consistent with the results of similar studies conducted in other countries (15, 18, 19), which found that endometriosis laparoscopy improved the SQOL of their participants; for example, the results of the Ferrero et al. study on postoperative SQOL showed that the frequency of intercourse increased 62.2% compared to the preoperative period, indicating a higher QOL and better sexual function in these women (18). However, this result was obtained in the follow-up period of 6 months, 1 yr, and sometimes 2 yr in the above studies, whereas we achieved this result in a shorter period (3 months) and the mean postoperative (6 months after surgery) severity of dyspareunia (1.44) in these women was significantly lower as compared to the preoperative phase (7.44).

In our study, there was an inverse correlation between SSE, SQOL and the stage of endometrial involvement; sexual satisfaction can decrease with the progression of the disease and its impact on dyspareunia. Ferrero et al. found that women in their study with higher endometrial involvement stages and endometriotic lesions of the uterosacral ligaments experienced more pain intensity (18). Jia et al. also concluded that sexual dysfunction was more common in women with endometriosis, especially in those with severe pelvic pain and advanced stages of endometriosis (26).

### Limitations

The limitations of this study included the difficulty in finding and following up participants after surgery and keeping track of the pathology results of the participants, which necessitated frequent contacts (by telephone and in person) with the participants. In this study, participants may have avoided providing the correct information about their symptoms and frequency of sexual intercourse, because of cultural reasons. Moreover, as sexual relations involve a mutual relationship and the sexual function of men is likely to be influenced by the sexual function of their wife, it is recommended that future studies also ask about male sexual function and SQOL.

## 5. Conclusion

Laparoscopic surgery for endometriosis can improve SQOL and SSE in affected women and can improve dyspareunia. Given the relationship between SSE and SQOL in participants with dyspareunia and due to the multifactorial nature of sexual function, it seems that the application of psychosexual therapy approaches should be considered by the healthcare team to improve the treatment process. Since this was a cross-sectional study, further studies such as case-control studies are recommended to evaluate the results with a control group.

##  Acknowledgments

The authors would like to express their thanks to Tarbiat Modares University, Tehran, Iran for its financial support of the present study, which is the result of a Master's thesis with the code 76946, as well as the women participating in the study and Mr. Ahmad Heydari for their sincere cooperation.
